# Performance of a new quantitative computed tomography index for interstitial lung disease assessment in systemic sclerosis

**DOI:** 10.1038/s41598-019-45990-7

**Published:** 2019-07-01

**Authors:** Marialuisa Bocchino, Dario Bruzzese, Michele D’Alto, Paola Argiento, Alessia Borgia, Annalisa Capaccio, Emanuele Romeo, Barbara Russo, Alessandro Sanduzzi, Tullio Valente, Nicola Sverzellati, Gaetano Rea, Serena Vettori

**Affiliations:** 1 Respiratory Medicine Unit, Department of Clinical Medicine and Surgery, Federico II University, Naples, Italy; 2 Department of Public Health, Federico II University, Naples, Italy; 3 Department of Cardiology, Monaldi Hospital - University of Campania “Luigi Vanvitelli”, Naples, Italy; 4 Rheumatology Unit, Department of Precision Medicine, University of Campania “Luigi Vanvitelli”, Naples, Italy; 5 Department of Radiology, Monaldi Hospital, Naples, Italy; 6 Section of Radiology, Unit of Surgical Sciences, Department of Medicine and Surgery, University of Parma, Parma, Italy

**Keywords:** Diagnostic markers, Systemic sclerosis

## Abstract

Quantitative high resolution computed tomography (HRCT) may objectively assess systemic sclerosis (SSc)-interstitial lung disease (ILD) extent, using three basic densitometric measures: mean lung attenuation (MLA), skewness, and kurtosis. This prospective study aimed to develop a composite index - computerized integrated index (CII) – that accounted for MLA, skewness, and kurtosis by means of Principal Component Analysis over HRCTs of 83 consecutive SSc subjects, thus eliminating redundancies. Correlations among CII, cardiopulmonary function and immune-inflammatory biomarkers (e.g. sIL-2Rα and CCL18 serum levels) were explored. ILD was detected in 47% of patients at visual HRCT assessment. These patients had worse CII values than patients without ILD. The CII correlated with lung function at both baseline and follow-up, and with sIL-2Rα and CCL18 serum levels. The best discriminating CII value for ILD was 0.1966 (AUC = 0.77; sensitivity = 0.81 [95%CI:0.68–0.92]; specificity = 0.66 [95%CI:0.52–0.80]). Thirty-four percent of patients without visual trace of ILD had a CII lower than 0.1966, and 67% of them had a diffusing lung capacity for CO <80% of predicted. We showed that this new composite CT index for SSc-ILD assessment correlates with both lung function and immune-inflammatory parameters and could be sufficiently sensitive for capturing early lung density changes in visually ILD-free patients.

## Introduction

Interstitial lung disease (ILD) is a leading cause of disability and mortality in systemic sclerosis (SSc)^1,2^, a chronic multiorgan disease characterized by autoimmune phenomena, microangiopathy, and fibrosis of the skin and internal organs^3–5^.

High resolution computed tomography (HRCT) detects SSc-ILD in up to 90% of cases^6–8^. In 2008, Goh *et al*. proposed a visual scoring of the total extent of SSc-ILD at HRCT that improved the prediction of mortality at follow-up (FU)^9^. These results were also confirmed by subsequent studies^10,11^.

However, this visual scoring of the extent of SSc-ILD remains affected by significant intra- and inter-observer variability^12,13^. Basic density-based CT analysis, such as mean lung attenuation (MLA, average global attenuation value of lung parenchyma), skewness (degree of histogram asymmetry) and kurtosis (degree of histogram peakedness), is widely available and could replace the visual scoring for SSc-ILD assessment^14–16^. This method also showed higher sensitivity as compared to the visual scoring^15^. Furthermore, densitometric parameters correlate with several functional indexes, such as forced vital capacity (FVC), diffusing lung capacity for carbon oxide (DLCO), and oxygen desaturation during the 6-minute walking test (6-MWT), and quality of life^15,17,18^.

In order to develop a single composite densitometric index for SSc-ILD quantification integrating MLA, skewness and kurtosis, we investigated a prospective series of SSc patients by low-dose thin section volumetric lung CT and searched for associations of this index with cardiopulmonary function parameters, and circulating markers of immune system activation (e.g. soluble interleukin-2 receptor alpha - sIL-2Rα - and chemokine CCL18) previously implicated in SSc and in SSc-ILD progression^19–22^.

## Materials and Methods

### Patients

SSc patients meeting the American College of Rheumatology/European League Against Rheumatism classification criteria^23^ consecutively visited at our outpatient clinic from July 2014 to July 2015 and giving written informed consent to the study were enrolled. Demographics, clinical and laboratory data instrumental to subset classification, that is either limited (lc) or diffuse (dc) cutaneous SSc^24^, and to organ/system involvement assessment according to international requirements^25^ [Supplementary Table S1], were collected. SSc specific autoantibodies were searched as previously described^26^. Disease duration was evaluated from Raynaud’s phenomenon (RP) onset. Spirometry, lung volume measurements and determination of the haemoglobin (Hb)-adjusted single-breath DLCO were performed at baseline and one year later using a computer-assisted spirometer (Quark PFT, Cosmed), according to international standards^27–29^. The 6-MWT was performed by trained hospital staff according to guidelines^30^. Standard echocardiography and Tissue Doppler Imaging were performed with a commercially available equipment (Philips iE33 ultrasound machine, Philips Medical Systems, Andover, MA) and a 2.5- or 3.5-MHz transducer by two highly trained cardiologists (MD and PA)^31^, according to international recommendations^32^. Patients with pulmonary hypertension (PH) and chronic obstructive pulmonary disease (COPD) were excluded from the analysis.

Serum levels of sIL-2Rα and CCL18 were measured by suspension immunoassays (Merk Millipore, Billerica, MA, USA) and read by a double laser-based fluorimetric instrument (Luminex 200, Luminex Corporation, Austin, TX, USA), according to manufacturer instructions.

We conducted our study in compliance with the principles of the revised Declaration of Helsinki and the study protocol was approved by the “Seconda Università degli studi di Napoli- Azienda Ospedaliera Universitaria SUN- AORN OSPEDALE DEI COLLI” Ethics committee (protocol n. 407/July 17^th^ 2014).

### Low-dose volumetric HRCT

Low-dose volumetric HRCT examinations were obtained with both a 16 slice multi-detector CT scanner (MDCT 16 Brilliance Philips, Eindhoven, the Netherlands) and a 64 slice multi-detector CT scanner (MDCT 64, General Electric Medical System, Milwaukee, WI), with patients in supine position, at full inspiration^33–35^. Scanning parameters were 120 kV and 80 mAs, by applying the smallest field of view according with the to patient body habitus. Matrix size was 512 × 512 pixels; images were reconstructed with a 1/1.25 mm slice thickness using bone filters. The whole chest volume was processed and stored on a picture archiving and communication system for post-processing evaluation (Dicom images). Lung parenchyma was independently analysed by two ILD-expert radiologists (GR and TV) with a window width of 1.600 Hounsfield Units (HU) and level −600 HU. The total ILD extent was visually assessed using the scoring system proposed by Goh *et al*.^9^. MLA, skewness, and kurtosis were calculated using a free open-source software for digital image processing (Image J, 1.51 I version, developed by the National Institutes of Health of the United States). Sampling of the whole lung volume was performed on axial HRCT images, after that anatomical structures that could lead to errors in the assessment of lung parenchyma density, such as trachea, bronchi and additional areas of the chest wall, were manually excluded as previously reported^14,36^. By digital image processing MLA, skewness, and kurtosis were then computed slice by slice and automatically generated averaged values from all slices were used for the development of a single index.

### Computerized integrated index development

MLA, skewness and kurtosis were combined in a single computerized integrated index (CII) using Principal Component Analysis (PCA)^37^. PCA is a data reduction technique where *p* variables measured on *n* subjects are combined to determine a new set of variables, called Principal Components, each of which represents a linear combination of the original variables and allows to explain a maximum but decreasing quota of the variability enclosed in the original dataset. In case of highly correlated variables (as MLA, skewness and kurtosis are), the first Principal Component accounts for the maximal amount of total variance in the observed variables and can be used to parsimoniously express the latent information shared by all of them thus discarding any redundancy. Each component is expressed by a set of *p* weights, one for each of the original variables, that is used to compute the subjects’ score on these new variables according to the following expression:

$${}_{{\rm{j}}}{\rm{C}}_{{\rm{i}}}{=}_{{\rm{j}}}{{\rm{b}}}_{1}{{\rm{x}}}_{1{\rm{i}}}{+}_{{\rm{j}}}{{\rm{b}}}_{2}{{\rm{x}}}_{2{\rm{i}}}+\ldots +,{{\rm{b}}}_{{\rm{i}}}{{\rm{x}}}_{{\rm{pi}}}$$

where _j_C_i_ is the score on the j-th component (j = 1, ..., n) for the i-th subject (i = 1, …, n), x_1i_,… x_pi_ are the values of the i-th subject on the *p* original variables and _j_b_i_,… _j_b_p_ represent the weights associated to the j-th component. The CII is, therefore, an a-dimensional index as in the PCA the components were standardized in order to give all of them equal weight in the data analysis.

The CII explained the 93.8% of the total variability. With respect to the original variables, this component showed a negative correlation with MLA (r = −0.94) and was positively correlated with skewness (r = +0.99) and kurtosis (r = +0.97). This correlation pattern allowed the assignment of a meaningful interpretation to the index, e.g. the lower the value of the CII is, the more severe the lung involvement is.

### Statistical analysis

The statistical platform R (The R Foundation for Statistical Computing) was used for all statistical analyses. Numerical variables were synthesized using mean ± standard deviation (SD) or, in case of consistent asymmetry in their distribution, using median with either range or inter-quartile range (IQR). Categorical variables were summarized using absolute frequencies and percentages. Differences between groups were accordingly assessed using either the *t* test for independent samples or the Mann Whitney U test in case of numerical variables and the chi-square test, or the Fisher exact test when appropriate, in case of categorical factors.

General linear models or logistic regression were used to adjust the analysis for confounding factors. Correlation and partial correlation were based on Pearson correlation coefficient.

Concordance between radiologists was measured using the Concordance Correlation Coefficient^38^ for quantitative measurements or by the Cohens’ Kappa index for dichotomic assessment. Diagnostic accuracy of the CII was measured using the area under the curve (AUC) of the corresponding receiver operating characteristic curve. The threshold for optimal classification accuracy was selected according to the maximization of the Youden Index.

## Results

### SSc patient characteristics

A total of 83 patients (79 females; mean age 56.4 ± 11.3 years) were enrolled in the current study. Seventeen (20.5%) had dcSSc and 66 (79.5%) lcSSc, with a median disease duration of 12 years (range 2–54). At visual assessment, ILD was detected in 39 (47%) patients, with a non-specific interstitial pneumonia (NSIP) pattern in 36 (43.4%) and a usual interstitial pneumonia pattern (UIP) in 3 (3.6%). Twenty out of the 39 (51.3%) SSc-ILD patients had an extensive disease according to the Goh score (e. g. >20%) with a 100% agreement between the two radiologists. Differences between patients with and without ILD in demographic, serological and clinical features are summarized in Table 1, with the exception of lung function that is detailed in Table 2. As expected, patients with ILD were mostly dcSSc, anti-topoisomerase-I antibody (ATA) positive with a shorter disease duration, and were more frequently treated with low-dose glucocorticoids and/or immunosuppressants (Table 1). Moreover, they had higher sIL-2Rα and CCL18 levels (Table 1), impaired lung function, and higher oxygen desaturation under effort (Table 2). There were no differences in terms of cardiac function between subjects with ILD and those without ILD (Supplementary Table S2), with the exception of an estimated systolic pulmonary artery pressure that was slightly higher in patients with ILD (30 mmHg [IQR 28.5–35] versus 28 [25–31.5]; p = 0.02).

**Table 1:** Characteristics of the SSc patients at baseline.

Characteristics	All patients (n = 83;%)	ILD+	ILD−	p*
		(n = 39;%)	(n = 44; %)	
***Demographics***				
Female	79 (95)	37 (94.87)	42 (95.45)	1
Age in years (mean±SD)	56.4 ± 11.3	56.51 ± 11.77	56.58 ± 10.61	0.979
BMI (median, range)	26 (19–40)	28 (19–40)	25 (20–39)	0.06
History of or current smoking attitude	41 (49.4)	14 (35.89)	27 (61.36)	0.028
***Disease subtype and clinical manifestations***				
lcSSc	66 (79.5)	25 (64.1)	41 (93.2)	
dcSSc	17 (20.5)	14 (35.9)	3 (6.8)	0.002
Years from 1^st^ non-RP symptom (median, range)	12 (2–54)	9 (2–49)	14 (2–54)	0.018
ANA	83 (100)	39 (100)	44 (100)	—
ACA	33 (39.76)	3 (7.69)	30 (68.18)	<0.001
ATA	35 (42.2)	32 (82.05)	3 (6.82)	<0.001
Anti-RNA pol III Ab	2 (2.4)	2 (5.13)	0	—
Anti-Pm-Scl Ab	0	0	0	—
Anti-fibrillarin Ab	0	0	0	—
Anti-Th/To Ab	0	0	0	—
Negative SSc-marker Ab	13 (15.66)	2 (5.13)	11 (25)	0.016
RP	83 (100)	39 (100)	44 (100)	—
History of and/or active DU and/or pitting scars	32 (38.55)	19 (48.72)	13 (29.55)	0.11
mRSS (median, range)	2 (0–28)	3 (0–26)	1 (0–8)	0.001
Joint/tendon involvement	37 (44.58)	19 (48.72)	18 (40.91)	0.513
Muscle involvement	3 (3.61)	1 (2.56)	2 (4.55)	1
Gastrointestinal involvement	59 (71.08)	27 (69.23)	32 (72.72)	0.81
Kidney involvement	0	0	0	—
***Circulating markers of immune system activation***				
sIL2-Rα (pg/ml; median, range)	125.4	161.5	110.1	<0.001
	(59.2–394)	(78–394)	(59.2–234.5)	
CCL18 (pg/ml; median, range)	0.056	0.073	0.045	0.002
	(0.01–1)	(0.01–1)	(0.01–0.32)	
***Therapies***				
GCs (≤10 mg/day in PDN equivalent)	51 (61.4)	35 (89.74)	15 (34.1)	<0.001
Previous cyclophosphamide	39 (46.99)	25 (64.1)	14 (31.82)	0.004
Current azathioprine	15 (18.07)	8 (20.51)	7 (15.9)	0.776
Current mycophenolate mofetil	30 (36.14)	23 (58.97)	7 (15.9)	<0.001
Low-dose aspirin	83 (100)	39 (100)	44 (100)	—
CCBs	83 (100)	39 (100)	39 (100)	—

Data are number and percentages (in brackets) except where otherwise indicated.

SSc = systemic sclerosis; ILD = interstitial lung disease; SD = standard deviation; BMI = body mass index; lcSSc = limited cutaneous systemic sclerosis; dcSSc = diffuse cutaneous systemic sclerosis; RP = Raynaud phenomenon; ANA = antinuclear antibodies; ACA = anticentromere antibodies; ATA = anti-topoisomerase-I antibodies; Anti-RNA pol III = anti-RNA polymerase III; Ab = antibodies; DU = digital ulcers; mRSS = modified Rodnan Skin Score; SRC = scleroderma renal crisis; HRCT = high resolution computed tomography; sIL2-Rα = soluble interleukin-2 receptor alpha; GC = glucocorticoids; PDN = prednisone; CCBs = calcium channel blockers.

*p is ILD+ versus ILD−.

**Table 2:** Lung function assessment in the SSc patients at baseline.

Parameter	All patients (n = 83)	ILD+ (n = 39)	ILD− (n = 44)	p*
Arterial pO_2_ (mmHg) at rest (21% FiO_2_)	83.7 ± 11.8	83 ± 2.2	84.2 ± 1.8	0.6
Arterial SpO_2_ (%) (median, range)	98.2 (83.5–100)	97.9 (83.5–99.5)	98.3 (95.3–100)	0.37
FVC (% predicted)	99.7 ± 25	89.2 ± 26.0	107.8 ± 21.2	0.001
TLC (% predicted)	80.9 ± 18.2	75.4 ± 20.9	85.6 ± 14.1	0.021
RV (% predicted)	78.5 ± 26.7	61.7 ± 3.6	91.7 ± 4.3	<0.001
DLCO_sb_ (% predicted)	59 ± 19.4	51.2 ± 18.9	65.4 ± 17.5	0.001
6MWT distance (mt) (median, range)	440 (176–566)	426 (264–528)	462 (176–566)	0.24
6MWT ΔSpO_2_ (%) (median, range)	2 (0–18)	3 (0–18)	1 (0–14)	0.002

Data are expressed as mean ± standard deviation, except where otherwise indicated.

SSc = systemic sclerosis; ILD = interstitial lung disease; pO_2_ = oxygen partial pressure; FiO_2_ = fraction of inhaled oxygen; SpO_2_ = oxygen saturation; FVC = forced vital capacity; TLC = total lung capacity; RV = residual volume; DLCO_sb_ = single breath diffusion lung capacity for carbon monoxide; 6MWT = six-minute walk test; mt = meters.

*p is ILD+ versus ILD−.

### CII correlations at baseline

**Figure 1:**
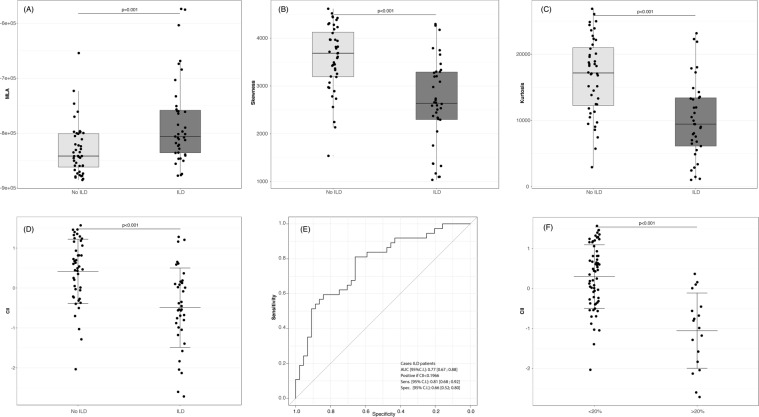
(**A**–**D**) Distribution of densitometric parameter values derived from the analysis of low-dose thin section volumetric lung CT in SSc patients without ILD in comparison with SSc-ILD cases. MLA, skewness and kurtosis are shown in panel (A–C), respectively. Distribution of CII values is shown in panel (D). The lower the value is for MLA and CII, the more severe the lung involvement is; on the contrary the higher the value is for skewness and kurtosis, the more severe the lung involvement is. (**E**) Diagnostic accuracy of CII by means of receiver-operating characteristic curve analysis. (**F**) CII in patients with limited (<20%) ILD in comparison with extended ILD (>20%), as assessed by the Goh visual score^10^. Lines represent mean ± standard deviation. CT = computed tomography; SSc = systemic sclerosis; ILD = interstitial lung disease; MLA = mean lung attenuation; CII = computerized integrated index; AUC = area under the curve; CI = confidence interval.

Density histogram analysis and CII evaluation are summarized in Fig. 1 (panels A-D). The CII discriminated between ILD and non-ILD SSc patients (p < 0.001), as patients with ILD has significantly lower CII values as compared to non-ILD patients (Fig. 1D). The best CII discriminating cut-off value for ILD cases was <0.1966 with a sensitivity and a specificity of 0.81 and 0.66, respectively, and an AUC of 0.77. The CII was also strongly associated with the Goh score (Fig. 1F).

**Figure 2:**
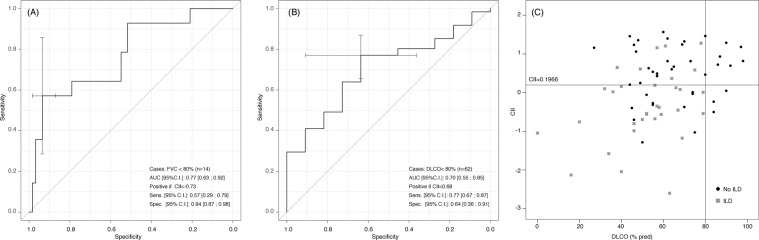
(**A**,**B**) Diagnostic accuracy of the CII according to baseline values of FVC (**A**) and DLCO (**B**) by means of receiver-operating characteristic curve analysis. (**C**) Scatter plot of CII and DLCO values stratified according the presence of ILD. 0.1966 was the cut-off value obtained by analyzing the diagnostic accuracy of the CII (see Fig. 1, panel E). CII = computerized integrated index; FVC = forced vital capacity; DLCO = diffusing lung capacity for carbon oxide; ILD = interstitial lung disease; AUC = area under the curve; CI = confidence interval.

**Figure 3:**
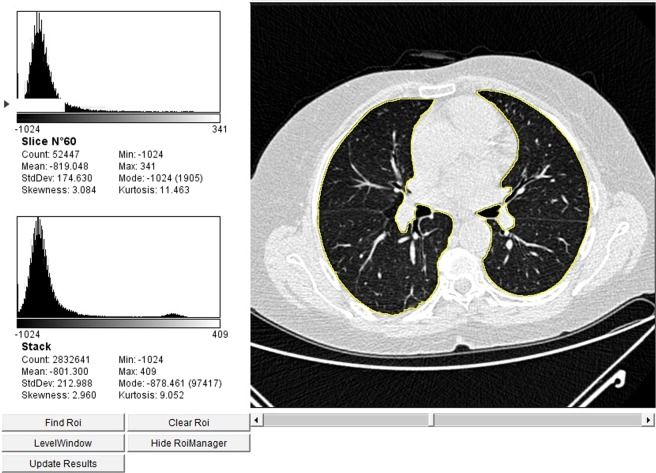
Comparison between quantitative and visual analysis of low-dose thin section volumetric lung CT in a SSc patient without visual evidence of ILD but a CII lower than the cut-off value of 0.1966 (e. g. CII = −0.3718). On the left, the figure shows the results from digital processing analysis: in the upper-left side of the image, data from the analysis of a single representative slice are given; in the lower-left side of the image the automatically generated averaged data from the analysis of all slices are given. Global pulmonary sampling has been performed and the sampled region is marked in yellow. CT = computed tomography; SSc = systemic sclerosis; ILD = interstitial lung disease; CII = computerized integrated index.

**Figure 4:**
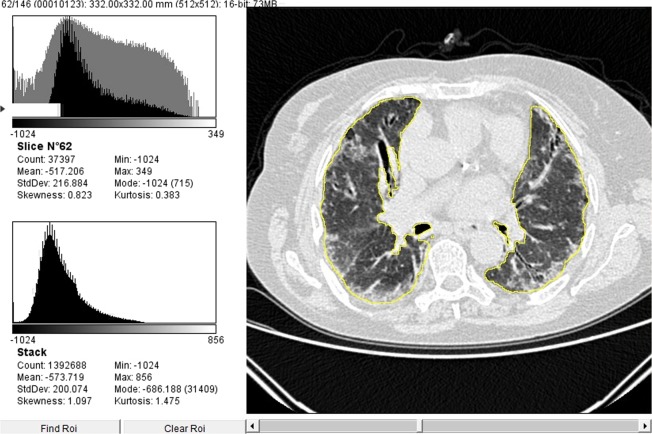
Comparison between quantitative and visual analysis of low-dose thin section volumetric lung CT in a SSc patient with ILD (Gho visual score >20%^10^; NSIP pattern) and a CII significantly lower than the cut-off value of 0.1966 (e. g. CII = −2.7150). On the left, the figure shows the results from digital processing analysis: in the upper-left side of the image, data from the analysis of a single representative slice are given; in the lower-left side of the image the automatically generated averaged data from the analysis of all slices are given. Global pulmonary sampling has been performed and the sampled region is marked in yellow. CT = computed tomography; SSc = systemic sclerosis; ILD = interstitial lung disease; CII = computerized integrated index; NSIP = non-specific interstitial pneumonia.

In keeping with the visual assessment (Table 1), the CII correlated with the body mass index (BMI) (r = −0.42; p < 0.001) and the disease duration (r = 0.22; p = 0.044); it was significantly lower in dcSSc as compared with lcSSc patients (−0.5144 ± 1.174 vs. 0.2277 ± 0.872; p = 0.019), in ATA positive subjects as compared with the other autoantibodies (−0.4049 ± 1.114 vs. 0.3249 ± 0.817; p = 0.008), and in subjects under previous or current treatment with cyclophosphamide (−0.2117 ± 0.972 versus 0.4165 ± 0.823; p = 0.003). Furthermore, the CII negatively correlated with sIL-2Rα and CCL18 serum levels (r = −0.27, p = 0.03; r = −0.34, p = 0.005, respectively), and with lung function and exercise performance parameters (Table 3). All of these differences remained significant after adjusting for BMI and disease duration. The CII accuracy in identifying patients with an FVC < 80% of predicted was of 0.77, the optimal cut-off being < −0.73 (57% sensitivity and 94% specificity) (Fig. 2A). The CII threshold of +0.68 differentiated patients with a DLCO < 80% of predicted with a sensitivity of 77% and a specificity of 64% (Fig. 2B). In 15/44 patients (34%) with no evidence of ILD at visual examination, the CII was lower than the cut-off value of 0.1966, and in 10/15 (67%) of them the DLCO was lower than the 80% of predicted (Fig. 2C). Figure 3 shows a representative CT scan from one of these patients where no ILD could be detected at visual assessment, but both the CII and the DLCO were lower than the respective cut-off values (e. g. −0.3718 and 70%). Figure 4 shows a representative case of visually detectable ILD (NSIP pattern, Goh score >20%) which was consistently associated with severe CII and DLCO reduction (e. g. −2.7150 and 39%, respectively).

**Table 3:** Correlations of the CII with lung function and exercise performance at baseline in the investigated SSc patients.

Parameter	Unadjusted	Adjusted*
pO_2_ (FiO_2_ 21%) at rest, mmHg	r = 0.28 (p = 0.01)	r = 0.19 (p = 0.09)
% SpO_2_ (at blood gas analysis)	r = 0.43 (p < 0.001)	r = 0.46 (p < 0.001)
FVC (% predicted)	r = 0.45 (p < 0.001)	r = 0.48 (p < 0.001)
TLC (% predicted)	r = 0.28 (p = 0.02)	r = 0.28 (p = 0.02)
RV (% predicted)	r = 0.52 (p < 0.001)	r = 0.45 (p < 0.001)
DLCO_sb_ (% predicted)	r = 0.34 (p = 0.03)	r = 0.36 (p = 0.002)
6MWT distance (mt)	r = 0.33 (p < 0.001)	r = 0.3 (p = 0.01)

^*^Adjusted for disease duration and Body Mass Index.

CII = computerized integrated index; SSc = systemic sclerosis; pO_2_ = oxygen partial pressure; FiO_2_ = fraction of inhaled oxygen; SpO_2_ = oxygen saturation; FVC = forced vital capacity; TLC = total lung capacity; RV = residual volume; DLCO_sb_ = single breath diffusion lung capacity for carbon monoxide; 6MWT = six-minute walk test; mt = meters.

### CII correlations at follow-up

At one-year, 52 SSc patients were re-assessed for lung function. The CII positively correlated with the total lung capacity (TLC) (r = 0.45, p = 0.004) and the DLCO (r = 0.29, p = 0.045), but not with the FVC, after adjustment for baseline values. Consistently, higher values of the CII were significantly associated with a decrease in the odds of a DLCO < 80% of predicted (Odds Ratio- OR = 0.19, 95% Confidence Interval- CI:0.05–0.73; p = 0.01) but not in the odds of an FVC < 80% of predicted (OR = 0.60, 95% CI:0.25–1.44; p = 0.25). Moreover, the CII was associated with a clinically significant DLCO decline (e. g. a DLCO decline ≥15%) in 7/52 (13.5%) of these patients (OR = 0.31, 95% CI:0.10–0.96; p = 0.042). Of note, although patients with a Goh score >20% had a three-fold risk of worsening DLCO, this association was not statistically significant (OR = 3.73, 95% CI:0.40 to 35.02; p = 0.249).

## Discussion

To our knowledge, this is the first prospective study that evaluated a composite index (the CII) incorporating several CT histogram metrics (e.g. MLA, skewness, and kurtosis) in the prediction of lung function deterioration over the short term FU.

Our novel CII strongly discriminated between SSc patients with ILD and those without ILD, with an excellent reproducibility (0.99) and an excellent correlation with each of its components, thus suggesting that it provides complete densitometric information eliminating redundancies. Notably, the CII also strongly correlated with the Goh assessment^9^, and was significantly lower in patients with an ILD extent >20% as visually assessed. In addition, the CII was associated with the main lung function parameters suggestive of a restrictive ventilation pattern, as with the DLCO and subcategories of the 6-MWT at baseline. These data are in line with previous studies that reported similar associations of lung function parameters in SSc with each of the lung density CT histograms that have been hereby used to elaborate the CII^16–18^.

We also found that a CII cut-off of 0.1966 identified the presence of ILD with a diagnostic accuracy of 0.77 and a sensitivity of 0.81 [95% CI:0.68–0.92] and a specificity of 0.66 [95% CI:0.52–0.80]. When we restricted the analysis of the CII to SSc patients with no visual evidence of ILD, we found that a significant proportion of these patients (34%) had a CII lower than the cut-off, and that the 67% of them had a DLCO lower than the 80% of the predicted value. These data suggest that this index may be more sensitive than the visual scoring and could be helpful for the earlier detection of ILD. In fact, the DLCO has been shown to be the best independent predictor of ILD progression in a recent large SSc cohort^39^, and in the idiopathic pulmonary fibrosis (IPF) setting^40^, even though this parameter is affected by different factors and it is not specific of fibrotic changes. However, since a major confounder of DLCO reduction in SSc is PH, our patients underwent a complete heart function evaluation to rule out PH cases.

We also found that the CII was significantly worse in patients with a longer disease duration, the dcSSc subset, and ATA positivity that has already been found to be predictive of ILD in large cohort studies^41,42^. Moreover, given the increasing amount of data providing evidence of novel circulating biomarkers of disease severity in SSc and of distinct SSc organ complications, we wanted to investigate the correlations of our index with serum levels of sIL-2Rα and CCL18. In details, we found that the CII correlates with both sIL2-Rα, that has been shown to reflect a more severe disease^19,20^, and with CCL18, that has higher serum levels in SSc patients with progressive ILD^21,22^.

Our study has some limitations. First, the study population size was not very large, and the prospective enrollment of consecutive patients did not allow any further enrichment of patients with early dcSSc. It is possible, in particular, that the long disease duration recorded in our patients (that is median 12 years from RP onset) affected a low risk to observe a more significant lung function deterioration during FU. However, a recent analysis from Khanna *et al*.^43^ pointed out that lung function deterioration over-time is similar in patients with a disease duration longer or shorter than 4 years.

The major strengths of our study are the prospective design, and the use of a low-dose volumetric chest CT protocol, that maintains both high sensitivity and lower radiation exposure for younger subjects. Interestingly, after adjusting the FU analysis for baseline values, the CII was still significantly correlated with the DLCO and the TLC at one-year, but not with the FVC. Most importantly, the CII predicted a clinically meaningful DLCO reduction in a subset of patients, while the Goh score did not. It is noteworthy that other Authors recently suggested that the quantitative analysis of HRCT density histograms could also be useful in assessing the risk of mortality in SSc-ILD^44^. Collectively, these observations suggest that the CII could be relevant to address some major unmet needs in the management of SSc-ILD. First, it catches very early lung modifications that occur before they become evident at visual HRCT examination, and could therefore help in identifying patients needing an early treatment. Second, it is an objective and sensitive tool that could be applied to the assessment of the scleroderma lung before and after treatment, thus revealing minimal changes that could be missed at visual evaluation. These two aspects are of utmost importance in clinical practice, since basing on data available in the literature not all SSc patients with ILD progress, and the need for treatment is made case by case^45^. Moreover, despite some open label studies and clinical trials have shown the benefit of immunosuppressive drugs in SSc-ILD, this benefit is limited to slowing down or halting progression with a non trivial treatment-related toxicity thus further affecting patients’ quality of life^46^. In this regard, Kloth *et al*. reported statistically significant lung texture changes at quantitative CT assessment in a retrospective series of 18/23 SSc patients who responded to autologous stem cell transplantation in terms of FVC stabilization or improvement after 6 and 12 months^47^, thus suggesting that quantitative CT analysis might be really useful in the assessment of the response to treatment. In another retrospective series, Kloth *et al*. also explored the potential utility of CT texture analysis in differentiating active alveolitis from lung fibrosis in SSc patients with ILD^48^. However, in both studies^47,48^ Kloth’s *et al*. used a number of niche, more sophisticated CT texture parameters (e. g. heterogeneity, intensity, average, deviation, entropy, uniformity, and contrast), that require advanced and not widely available analytic tools. Therefore, as the same Authors stated, the daily use of such advanced CT texture analysis in SSc-ILD is still not feasible, and needs further validation in prospective cohort studies along with CT protocol standardization. This means that such an approach is currently limited to specific research settings. On the contrary, MLA, skewness, and kurtosis can be easily computed with open-source softwares^16^, can be summarized in a single index, as shown here, and could be applied in the routine clinical practice to systematically screen for early lung involvement each SSc patient and to FU the disease. Moreover, the correlation of textural analysis parameters with the validated outcome measures of lung function in SSc appears to be weaker than the correlation shown by the three basic densitometric histograms (e.g. MLA, skewness and kurtosis) used in our study and in previous reports^49^.

In conclusion, in the context of a growing interest for new methods devoted to optimize SSc-ILD detection and quantification^15–18^, our new CII could be helpful in driving the choice of patients to treat in the context of a more defined precision medicine approach, which ultimately will improve survival and well-being based on individualized and tailored patient management. To this purpose, the potential application of the CII in low-dose volumetric HRCT protocols for the early quantitative detection of lung density changes in SSc patients should be explored in larger multicenter cohort studies.

## Supplementary information

Supplementary material

## Data Availability

The datasets generated during and/or analysed during the current study are available from the corresponding author on reasonable request.
