# Supplementary material for: Performance of a new quantitative computed tomography index for interstitial lung disease assessment in systemic sclerosis
Source: Sci Rep. 2019 Jul 1;9:9468. doi: 10.1038/s41598-019-45990-7 (PMC6603022; doi:10.1038/s41598-019-45990-7)
Supplement: Supplementary file 1 — Supplementary material [file 41598_2019_45990_MOESM1_ESM.doc]

**Title:** Performance of a new quantitative computed tomography index for interstitial lung disease assessment in systemic sclerosis.

**Running head:** A new quantitative CT index for SSc-ILD

**Author list:** Marialuisa Bocchino ^1†^, Dario Bruzzese ^2†^, Michele D’Alto ^3^, Paola Argiento ^3^, Alessia Borgia ^4^, Annalisa Capaccio ^1^, Emanuele Romeo ^3^, Barbara Russo ^4^, Alessandro Sanduzzi ^1^, Tullio Valente ^5^, Nicola Sverzellati ^6^, Gaetano Rea ^5†^, Serena Vettori ^4†*^

**Affiliations:** ^1^Respiratory Medicine Unit, Department of Clinical Medicine and Surgery, Federico II University, Naples, Italy; ^2^Department of Public Health, Federico II University, Naples, Italy; ^3^ Department of Cardiology, Monaldi Hospital - University of Campania “Luigi Vanvitelli”, Naples, Italy; ^4^Rheumatology Unit, Department of Precision Medicine, University of Campania “Luigi Vanvitelli”, Naples, Italy; ^5^Department of Radiology, Monaldi Hospital, Naples, Italy; ^6^Section of Radiology, Unit of Surgical Sciences, Department of Medicine and Surgery, University of Parma, Parma, Italy.

†These Authors contributed equally to the study

**Correspondence to:** Marialuisa Bocchino, Respiratory Medicine Unit, Department of Clinical Medicine and Surgery, Federico II University, Monaldi Hospital, Via L. Bianchi 1, 80131 Naples, Italy; e-mail: [marialuisa.bocchino@unina.it](mailto:marialuisa.bocchino@unina.it) & Serena Vettori, Rheumatology Unit, Department of Precision Medicine, University of Campania “Luigi Vanvitelli”, II Policlinico, Via S. Pansini 5, 80131 Naples, Italy; e-mail: [serena.vettori@unicampania.it](mailto:serena.vettori@unicampania.it); Orcid ID: 0000-0001-5617-9829.

**Supplementary table S1.** Core set variables to be assessed in SSc patients in clinical studies according to the European Scleroderma Study Group to define the presence and extent of organ involvement.

| **Organ/system** | **Feature** |
| --- | --- |
| *Skin*  *Peripheral vessels*  *Joint/tendons*  *Muscle*  *Gastrointestinal tract* | Modified Rodnan Skin Score  Raynaud’s phenomenon  Digital ulcers and/or pitting scars at fingerpad  Measurement of finger tip distance  DAS28 in case of synovitis  Tendon friction rubs  Proximal muscle weakness  Serum CK elevation  Esophageal and/or stomach and/or intestinal symptoms  Barium esophageal X-ray abormalities  Small bowel series abnormalities |
| *Lung*  *Heart*  *Kidney* | Dyspnea  Fibrosis at standard chest X-ray  Restrictive pattern at lung function test  Systolic pulmonary artery pressure elevation estimated on cardiac echoDoppler study  Symptoms of heart disease  ECG abnormalities (conduction defects, arrhythmias)  Pericardial effusion, impaired EF, inverted E/A ratio on cardiac echoDoppler study  New onset arterial hypertension  Serum creatinine elevation  Urinalysis abnormalities |

SSc = systemic sclerosis; DAS28= disease activity score on 28 joint count; CK= creatin kinase; ECG= electrocardiography; EF= ejection fraction; E/A= early/atrial (=late) ventricular filling velocity.

**Supplementary table S2.** Echocardiographic findings in SSc patients at baseline.

Data are expressed as median (range) or as mean ± standard deviation.

| **Parameter** | **All patients (n=83)** | **ILD+**  **(n=39)** | **ILD-**  **(n=44)** | **p*** |
| --- | --- | --- | --- | --- |
| *Standard echocardiography*  % EF  TAPSE, mm  sPAP (mmHg)  CI | 60 (25-71)  23.2 ± 3.7  30 (20-50)  2.9 ± 0.7 | 60 (40-71)  22.7 ± 4.3  30 (20-38)  2.9 ± 0.7 | 60 (25-68)  23.7 ± 3  28 (20-50)  2.8 ± 0.6 | 0.685  0.234  0.022  0.739 |
| *TDI*  S_m_ (cm/sec)  S_t_ (cm/sec)  E_m_/A_m_  E_t_/A_t_  E/e’ | 9 (5-21)  13 (8-24)  0.9 (0.4-5.3)  1.1 (0.5-1.9)  8 ± 2.6 | 9 (5-21)  14 (10-22)  1.1 (0.5-2.9)  1.1 (0.6-1.9)  7.8 ± 2.4 | 9 (5-13)  13 (8-24)  0.9 (0.4-5.3)  1.2 (0.5-1.9)  8.1 ± 2.8 | 0.988  0.267  0.912  0.347  0.531 |

SSc= systemic sclerosis; ILD= interstitial lung disease; EF= ejection fraction; TAPSE= tricuspid annulus plane systolic excursion; sPAP= systolic pulmonary artery pressure; CI= cardiac index; TDI= Tissue Doppler Imaging; S_m_= S wave at mitral annulus; S_t_= S wave at tricuspid annulus; E_m_= E wave at mitral annulus; A_m_= A wave at the mitral annulus; E_t_= E wave at tricuspid annulus; A_t_= A wave at tricuspid annulus; E= E wave on transmitral Doppler; e’= early relaxation velocity on Tissue Doppler.
